# The Role of the Public Health Workforce in Securing Political Commitment for Tackling Childhood Obesity in Local Government

**DOI:** 10.1177/15248399241294231

**Published:** 2024-11-18

**Authors:** Ravita Taheem, Kathyrn Woods-Townsend, Wendy Lawrence, Janis Baird, Keith M. Godfrey, Debbie Chase, Mark A. Hanson

**Affiliations:** 1 NIHR Southampton Biomedical Research Centre, University Hospital Southampton NHS Foundation Trust, Southampton, UK; 2 Southampton City Council, Civic Centre, Southampton, UK; 3 Southampton Education School, Faculty of Social Sciences, University of Southampton, Southampton, UK; 4 Medical Research Council Lifecourse Epidemiology Centre, University of Southampton, Southampton, UK; 5 Primary Care, Population Science and Medical Education, University of Southampton, Southampton, UK; 6 NIHR Applied Research Collaboration Wessex, Southampton Science Park, Southampton, UK; 7 Institute of Developmental Sciences, University of Southampton, Southampton, UK

**Keywords:** public health, health promotion, local government, childhood obesity, accountability, political commitment

## Abstract

Local government is well placed to provide leadership for the national ambition in England to halve childhood obesity by 2030 and has delegated authority for many policies thought necessary for effective action. However, with currently constrained local government finances, resources may not be prioritized for the wide range of policies and interventions necessary. Thus, local political commitment is vital for directing local government investment to tackle the issue. This qualitative study investigated how political commitment has been demonstrated in local government, the strategies used to build such political commitment, and the role of the public health workforce. Public health staff from 13 local authorities participated in semi-structured telephone interviews using questions informed by a conceptual framework composed of five previously defined levels of political commitment: rhetorical, institutional, operational, embedded, and system-wide. Thematic analysis showed that public health officers secured a political commitment by framing childhood obesity in line with existing priorities to build support among councillors in a position to advocate for the issue. Public health officers also engaged with multiple stakeholders, gathering data and insights to develop operational and embedded commitment. The study shows that effective health promotion practice requires a skilled and committed public health workforce, acting as policy entrepreneurs to secure system-wide political commitment in local government by proactively navigating local governance and policy processes, to build in opportunities for feedback and accountability.

In England one in five children starting primary school (aged 4–5 years) are overweight or obese, rising to one in three by the time they leave primary school (aged 10–11 years) (Office for Health Improvement and Disparities, 2022). The recent National Child Measurement Programme (NCMP) data for 2021-2022 showed widening disparities, with childhood obesity levels twice as high in the most disadvantaged areas compared with the least disadvantaged areas in England (NHS Digital, 2022). Disadvantage relates to the English indices of deprivation based on seven domains: income, employment, education, health, crime, barriers to housing, and the living environment (Ministry of Housing, 2019). In Southampton pooled NCMP data from 2019–2020 to 2021–2022 for school children aged 10 to 11 showed 43.7% from the most disadvantaged areas were living with excess weight (overweight and obesity) compared with 32.0% in the least disadvantaged areas (Southampton Data Observatory, 2023). Childhood obesity is linked to greater risks of later chronic diseases, especially diabetes, cardiovascular disease, asthma, muscle and joint problems, and some types of cancer (Bhutta et al., 2023; Horesh et al., 2021). Childhood obesity can also have an immediate impact on the lives of children and young people as it is associated with lower self-esteem, behavioral problems, and lower educational attainment (Rankin et al., 2016; Sanders et al., 2015; Segal et al., 2021). Also, the costs of obesity to individuals, and the socio-economic costs include increased health care costs, loss of productivity, sickness absence, increased mortality before retirement, and early retirement due to poor health (World Obesity Federation, 2023).

Effective health promotion practice will be critical in achieving the national ambition in England to halve the prevalence of childhood obesity by 2030 (Department of Health and Social Care, 2018). However, an analysis of government strategies for addressing obesity found that, except for the soft drinks industry tax, simple information-giving and capacity development have been prioritized and will likely be ineffective (Theis & White, 2021). More recent regulations to restrict the placement of food and drink high in fat, sugar, and salt by volume (e.g., “buy one get one free”) signal some action has occurred, but the pace remains inadequate (Department of Health and Social Care, 2022).

The World Health Organization in their Report of the Commission on Ending Childhood Obesity noted that the lack of political commitment is “the greatest obstacle to effective progress on reducing childhood obesity” (World Health Organization, 2016, p. XIII). Political commitment has been described as generating interest and getting the issue on the political agenda, adopting policies, mobilizing institutions, allocating resources and coordinating actions (Baker, Hawkes, et al., 2018; Pelletier et al., 2011). Thus, gaining political commitment can lead to the support and resources needed to implement and sustain interventions (Baker et al., 2019). Local authorities in England have a mandate to promote population health, although there is no specific mandate to address childhood obesity. However, without local investment, the national ambition to halve childhood obesity is unlikely to be achieved.

The limited political commitment may be partly due to challenges in framing an issue with multifactorial causes. Studies have found that policy attention to the social determinants of health is constrained due to the multiple causes and the lack of feasible, evidence-based solutions (Baker, Friel, et al., 2018). Furthermore, policy researchers refute the common assumption that knowledge and evidence alone directly shape policy, showing that policy-makers often make selective use of research evidence, drawing on gut instinct, personal values, and previous positions in their decision-making (Cairney & Oliver, 2018).

Studies on the political commitment to nutrition in low or middle-income countries provide a useful parallel for exploring childhood obesity at the local government level. These studies have found that high levels of expressed commitment from political leaders may not lead to policies or resources to implement change. This highlights the requirement for a broader understanding of political commitment, which goes beyond just attention and verbal commitment, to include resources and the infrastructure to support implementation (Fox et al., 2014; Pelletier et al., 2011).

Nutrition actor networks (individuals and organizations with a shared interest in reducing malnutrition) have been crucial to developing political commitment by providing advocacy and offering technical expertise (Baker et al., 2019; Baker, Hawkes, et al., 2018). Similar to nutrition actors, policy researchers describe “policy entrepreneurs” (government officials or external actors, but not politicians) as advocates for the issue, with an understanding of the political landscape to support agenda setting and policy design (Heaver, 2005).

## Purpose

In England, local authority public health officers have been encouraged to lead a whole systems approach to influence a wide range of health promotion policies to prevent obesity. However, little is known about political buy-in for tackling the issue in local government. Therefore, this study aimed to apply the conceptual framework drawn together by Baker, Hawkes, et al. (2018) to characterize how political commitment to tackling childhood obesity is demonstrated in local government to support local action, as perceived by local public health officers. The study also aimed to explore the strategies adopted by the local public health workforce to develop political commitment.

## Method

### Study Design

A qualitative study was undertaken, involving semi-structured telephone interviews with public health practitioners/ managers and public health consultants. Ethics approval was granted in March 2019 from the Faculty of Medicine Ethics Committee at the University of Southampton (ERGO 46896).

### Participants

Public health officers with a lead role in healthy weight, obesity, or childhood obesity in local authorities classed as statistical neighbors and Office of National Statistics (ONS) comparators for Southampton City Council were invited to participate. The term “Statistical Neighbor” describes local authorities grouped based on a range of variables derived from census data, and is used in practice for the purposes of benchmarking (Department for Education, 2021). Statistical neighbors for children’s service data and ONS comparators for Southampton are routinely updated by Southampton City Council.

### Recruitment

The Directors of Public Health for all 19 local authorities classed as statistical neighbors or ONS comparators for Southampton were approached via email. The email described the purpose of the study and requested participation from the relevant officer. A second email was sent to those who did not reply.

Due to some non-responses from statistical neighbors, an invitation to participate in the research was sent to 17 authorities in the region. These included unitary authorities (which provide all local government services) and two-tier authorities (which divide local government services between county councils and smaller district councils). They were contacted via the South-East Healthy Weight Network of healthy weight lead officers organized by Public Health England (functions now included in the government Office for Health Improvement and Disparities (OHID)). A table of local authorities invited is provided as a supplemental file (Supplemental File 1).

### Theoretical Framework

Baker, Hawkes et al. (2018) drew together previous research and described five levels of political commitment (rhetorical, institutional, operational, embedded, and system-wide) for action on nutrition, which provides a useful conceptual framework for this study. Rhetorical commitment can be expressed in the form of statements made by the political and legislative branches of government. Institutional commitment is indicated by the presence of a policy infrastructure that allows the adoption of legislation or policies. Operational commitment describes the allocation of resources (financial, human, technical) to allow action on the ground. Embedded commitment is the work of other departments which indirectly contributes to addressing the issue. System-wide commitment has been described as sustained and institutionalized policy responses, with a “positive feedback loop” to enable long-term action (Baker, Hawkes, et al., 2018). This framework was used to inform the semi-structured discussion guide and to categorize the data in the initial stage of the qualitative analysis.

## Materials

A semi-structured questionnaire and discussion guide were produced based on the five levels of political commitment conceptual framework (Table 1; Baker, Hawkes, et al., 2018). All participants were sent an information sheet describing the study and a consent form before the interview. Telephone interviews were undertaken by the lead author, a senior public health practitioner and post-graduate researcher. Interviews took place between August and December 2019 and were audio-recorded; the length ranged from 21 to 52 min (mean duration was 33 minutes).

**Table 1:** Semi-Structured Discussion Guide

*Questions and prompts*
Exploratory questions What are the key policy priorities for your council? What plan does your council have to tackle childhood obesity? Findings so far show that the main types of interventions had an individual focus rather than addressing the wider determinants of health. What are the main types of interventions used in your council to tackle childhood obesity?
Commitments made in public (rhetorical commitment) What written or spoken statements on childhood obesity have been made by your local councillors?
Action taken (institutional commitment) What action has been taken to tackle the issue? How has tackling childhood obesity been included in policies across your organization? What impact has that had?
Allocation of resources (operational commitment) What resources have been allocated to tackle childhood obesity? How have resources outside the public health grant been allocated to tackling the issue?
Embedded commitment What has been done to encourage other departments at the council to tackle childhood obesity? How do you think this has raised the profile of childhood obesity?
System-wide commitment How do local councillors and leaders get feedback on the policies and programs in place to tackle childhood obesity? How easily can the policies and programs adapt to the changing conditions? What other factors do you think could support a system wide commitment to tackling childhood obesity?
What have I missed, what else would you like to add before we end the interview?

### Data Analysis

The audio recordings were transcribed and reviewed for accuracy and the transcripts were coded using NVIVO 12 Pro qualitative data analysis software. Using a codebook the initial transcripts were coded deductively to the five levels of political commitment (Braun & Clarke, 2021a). The data were organized into the five main categories of political commitment, with additional sub-categories that included a description to summarize the content and meaning of the data. Two transcripts were discussed and double-coded with the involvement of a second coder and any disagreements were discussed until agreement was reached to support a reflexive analysis. The sub-categories required amendment and the data (all transcripts) were analyzed again using the revised sub-categories. Further inductive analysis was undertaken using the sub-categories to establish themes under each category addressing the research questions on how each type of commitment was demonstrated in local authorities and what strategies public health officers use to develop that commitment. At least 6 participants spoke about each of the described themes. The initial candidate themes were reviewed against categorized extracts of the raw data to ensure that they reflected the overall dataset and were discussed with other researchers before they were finalized.

Once the initial data analysis had been completed, the interpretation of the results was presented in the form of a causal loop diagram and sent to participants as a respondent validation exercise. This was developed following guidance on using qualitative data to build a causal map and involved creating word-and-arrow diagrams to illustrate the relationship between different actors and the five levels of political commitment (Kim & Andersen, 2012). For example, the analysis showed that public health officers regularly raise the issue of childhood obesity with councillors (elected local government officials), which was illustrated with arrows to show “policy entrepreneur” time or “operational commitment” was required to engage a “political actor.” Heaver (2005) described three groups of actors: (a) champions—politicians and decision-makers who directly influence policy; (b) entrepreneurs—civil servants and researchers who can influence champions; and (c) supporters—broadly involved but not in strategy development or bringing together stakeholders. Participants were sent the causal loop diagram with a short description of the five forms of political commitment and examples of the role of each category of actor. Participants were asked: (a) if they understood the causal loop diagram and how it could be improved, and (b) whether it reflected their reality, and if not, what would they change?

## Results

A public health officer (including directors, public health consultants, practitioners, and managers) representing ten statistical neighbor local authorities agreed to participate in the research: Nottingham, Birmingham, Coventry, Derby, Portsmouth, Bristol, Leeds, Plymouth, Hull, and Southend-on-Sea. In addition, three public health officers from Slough, Medway, and Kent participated.

Opportunities for political commitment to tackling childhood obesity are available in local government, using public platforms, via strategies, and through wider council functions. However, the data indicate that childhood obesity is perceived as a difficult topic for councillors to engage with. Therefore, it requires careful facilitation by public health officers through discussions, using opportunities provided through the existing council democratic and governance infrastructure, as well as through engagement with stakeholders. The themes are discussed below with illustrative quotes for each theme in Table 2.

**Table 2:** Categories, Themes, and Quotes

*Categories*	*Themes*	*Quotes*
Rhetorical commitment	Rhetorical commitments made in existing platforms	ID11: “So whether it is discussions at Health Scrutiny, Health and Wellbeing Board, all the different levels, they are now showing an active interest in what we as a system are doing towards childhood obesity.” ID05: “. . . we did the sharing event. So at that event the Council Leader, both the Executive Lead members for Children and Health and Wellbeing all attended to say “hello” to all of the delegates and to talk about how important this issue was from their perspective.”
	Secured through aligning the issue with councillors’ priorities	ID04: “. . . our cabinet member who is prepared to stick her neck out really and take this through. Largely supported by the fact that we’re going for the Sustainable Food City award which currently nobody has . . . I think that was the thing that enabled her to show the amount of support. . .”
Institutional commitment	Inclusion in council strategies and advocates among decision-makers	ID03: “. . . there’s a commitment to reduce childhood obesity by 10%; that will be in the upcoming council plan and it was certainly in the last council plan around childhood obesity as well.” ID11: “. . . what we do have is really good buy-in at political level, so our councillor portfolio holder for health and social care has been an absolute advocate of the work that we do. . . she understands the wider impact that it has on the community.”
	Strategic officer advocates and provides reassurance about activities	ID11:“. . . our consultant, she sits on the various director level boards and Senior Leadership Team or Department Management Team. Her responsibility is to feedback the work that we are doing, but she has been really pivotal in doing that work and actually engaging other people and getting them to understand the importance of what we are doing.”
Operational commitment	Resources committed or leveraged from other departments	ID04: “We’ve had to decommission lots of our services that weren’t statutory. So we don’t have a weight management commissioned service anymore.” ID10: “We have got an environmental health team that are doing healthy option schemes for local takeaways. We have our town planners looking at restricting takeaways and what else they can do. We have got some fantastic parks and open spaces across the city, so significant investment. We have got highways looking at active travel.”
	Developing partnerships, gathering data and insights	ID02: “Okay, so it’s worth saying that the Health and Wellbeing Board chose childhood obesity as one of its priorities this year and then through the workshop that we did with partners, we got to healthy food city and active city so actually through ‘workshopping’ we did that. ID02: We’re now in a space about going- what are the things that would really drive a system conversation and close the data gap that we’ve got about food in the city? Because we know very little about the food environment”
Embedded commitment	Departments outside public health indirectly engaged	ID03: “So we start with the cross over areas and go from there you know. Air pollution is a massive issue in [x city] and we’ve done a lot on clean air, and actually that really drives our agenda around active travel and physical inactivity, finding those links is really important to get that buy in.”
	Routine practice of other departments has been influenced	ID12: “We work with our planning team. . . So, we are looking to convert our guidance note into full policy, around restricting access to hot food takeaways. So, you will find at least five or six departments having some actions in their service plan which is the thing that drives annual activity.”
System-wide commitment	Reporting using council governance mechanisms	ID05: “So whenever they want to have an update about what’s going on, we can go to the Health and Wellbeing Board. In addition, we might go to Scrutiny [Panel] if they are interested, we’ve talked to them. We go to the community committees sometimes, and talk about the work as well. So we use a variety of different routes to really share and update people about what’s working.”
	Governance and review are proactively sought	ID10: “So, first of all I think they must have asked for a report about three years ago on childhood obesity. I don’t know where the original request came from, but in my papers each one I have ended with, I recommend the next steps is we continue the whole system approach and that Health Scrutiny (to keep the political pressure and leadership) recall this next year for an update. And then that just automatically gets put on and then in a year’s time you get hauled up again.”

### Political Commitment Is Demonstrated Through Strategies, Investment by Other Council Functions, and Through Formal and Informal Discussion Platforms

Institutional commitment to tackling childhood obesity was demonstrated through departmental or overarching council-wide strategies, or through stakeholder sign-up to an agreement or pledge to ensure that their activities and decisions considered the impact on childhood obesity. Both of these mechanisms provide public-facing documentation of political commitment. This was strengthened in some cases through the support of a political champion with an understanding of the wider implications of childhood obesity and who could advocate for the issue.

Some participants noted operational commitment through commissioned services (services that are designed, purchased, and monitored for a local population; NHS England, 2024), but others reported limited funding and competing priorities, meaning that lifestyle services (services supporting lifestyle changes for weight loss) had to be decommissioned. Particularly prominent was the role of other council departments. Participants frequently shared how activities and projects undertaken by council departments such as Planning, Transport, Economic Development, Education, and Licensing, contributed to addressing and preventing childhood obesity. Much of the work undertaken was indirect, where other agendas such as air pollution and active travel drove the activity, illustrating how embedded commitment from departments such as Planning and Transport could indirectly influence childhood obesity.

Both rhetorical commitment and system-wide commitment to tackling childhood obesity were facilitated by council democratic and governance infrastructure. Rhetorical commitment was expressed in formal council platforms such as Health and Wellbeing Boards (a statutory committee in local government, with leaders from the health and care system to improve health and well-being and reduce health inequalities for their local population), and Scrutiny committees (Health overview and scrutiny committees are a local government statutory function to ensure local health service bodies are accountable to their communities) led by councillors, and in informal settings through council or external partnership forums and events. In some cases, these opportunities were facilitated by officers, providing councillors with the platform to present or to promote new or existing local projects. Similarly, system-wide commitment was demonstrated in a limited way when activities and progress on childhood obesity were reported as part of a local authority’s governance structure, including the Health and Wellbeing Board, the Children’s Board or Community Boards. Participants frequently highlighted that system-wide commitment was limited by a lack of coordination between the national and local government policy agendas, particularly in relation to the food and drink industry, where there is limited opportunity to influence policies at a local level.

### Political Commitment Is Facilitated Through Careful Framing of the Issue, Influencing Practice Across Departments, and the Proactive Use of the Democratic Governance Infrastructure

Rhetorical and institutional commitment was facilitated by public health officers regularly raising the issue and framing it in a way that resonated with councillors. Childhood obesity was often co-framed with issues such as physical activity, active travel, and holiday hunger (describing an issue for school pupils who are eligible for free school meals during term time but may go hungry during the school holidays). To support institutional commitment, strategic officers were able to engage routinely with leadership at the council, and regularly raised the issue to update decision-makers. With careful framing, participants frequently stated the importance of providing reassurance about the progress being made to secure buy-in from leaders.

The data suggested that public health officers both drove and provided operational commitment in multiple ways, by supporting policy development through formal council structures and forums, such as the Health and Wellbeing Boards, and by developing partnerships with other council departments and sectors. A key priority for participants was identifying where gaps in the data lay and undertaking work to fill those gaps, which helped to shape local narratives about childhood obesity and its wider implications.

Participants described several ways that embedded commitment was facilitated: by contributing to the service plans and contracts of other departments; developing guidance documents or principles to enable other departments to consider the public health implications of their decisions; and by providing training or using health impact assessments or audits to support action. This informal support was not directly linked to policy development but describes how the practice could be influenced to secure embedded commitment.

Finally, public health officers were able to access existing governance structures to provide updates at a strategic level, although less often across the other organizations/ sectors, to support a system-wide commitment. Some participants described how they routinely requested review by decision-makers in key forums, so that progress on the issue was re-examined by local councillors. In this way, by securing a position on the decision-making agenda on a regular basis, the officers were routinely held accountable by leaders and decision-makers.

### Participant Validation

**Figure 1:**
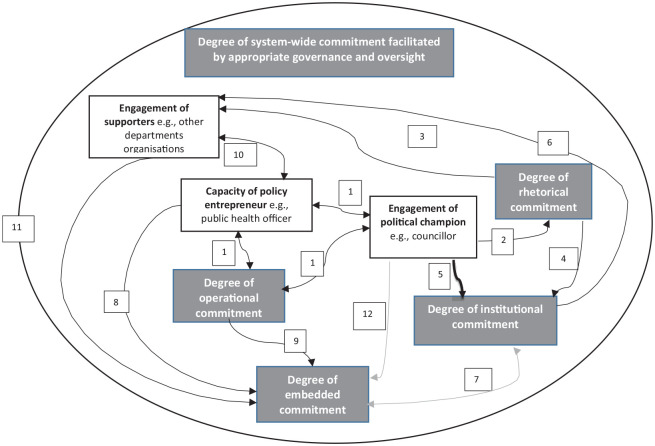
Causal Loop Diagram of Political Commitment for Tackling Childhood Obesity in Local Authorities *Note.* Each numbered point below corresponds to the numbered box in the causal loop diagram and describes the relationship represented by each arrow. Amendments were made as a result of feedback from participants represented by boxes 7 and 12. (1) The policy entrepreneur provides an operational commitment to engage political champions, the two-way arrows reflect ongoing dialogue. (2) The political champion can make rhetorical commitments internally at council forums and externally. (3) Rhetorical commitments can generate supporters in the local authority and externally. (4) Rhetorical commitments made by a political champion can lead to an institutional commitment from other councillors or leaders. (5) The political champion can prioritize the issue to strengthen institutional commitment. (6) Institutional commitment can generate supporters in the local authority and externally. (7) Institutional commitment enables embedded commitment from other departments. Embedded commitment can lead to institutional commitment. (8) The policy entrepreneur facilitates embedded commitment by working with other departments. (9) Operational commitment (financial resources) can lead to embedded commitment through commissioned work. (10) The policy entrepreneur engages with multiple stakeholders to develop supporters. Ongoing dialogue to gather insights/data. (11) Supporters among council departments can lead to embedded commitment and resources to indirectly tackle the issue. (12) The political champion can influence embedded commitment from other departments.

Ten out of 13 participants responded to the request for feedback on the causal loop diagram (Figure 1), which illustrated how actors could influence multiple forms of political commitment. Most participants reported that the findings reflected their reality. Two participants suggested relevant additional points: first, a political champion could influence embedded commitment from other council departments and second, policy entrepreneurs can influence institutional commitment more broadly as they have the duty and autonomy to generate buy-in from both internal and external stakeholders.

## Discussion

This study used an evidence-based conceptual framework on the five levels of political commitment to deepen understanding of political commitment for health promotion policies to tackle childhood obesity in local government in England. Specifically, this study gained perspectives from local government officers, who have a role in driving the agenda for their local authority. This research revealed how public health officer-led actions could contribute to developing multiple types of political commitment within local systems and underlined the importance of a highly skilled workforce able to align with councillors’ priorities, support the development of political champions, and navigate the local democratic infrastructure.

Our research indicated that officers perceived childhood obesity as a difficult topic for councillors to engage with, therefore, the issue was often co-framed with other priorities, as a way of gaining political attention. This finding is similar to research on nutrition in low or middle-income countries (Baker et al., 2019; Pelletier et al., 2011). Existing research cautions that multiple framings of an issue may “cloud perceptions” and confuse policy solutions (Balarajan & Reich, 2016). However, co-framing may be important initially, before stronger political engagement can be established.

This study showed that formal Health and Wellbeing Boards and Scrutiny Committees, as well as informal events, provide opportunities for rhetorical commitment and that these opportunities can be facilitated by officers. Although rhetorical commitment such as a verbal statement of support from a councillor may not lead to policies or resources, it provides an opportunity for the problem to be documented, which may encourage policy intention (Pelletier et al., 2011). Institutional commitment was enhanced when councillors took on the role of advocate in addition to having organizational strategies. However, the lack of data in this study about how rhetorical and institutional commitment contributed to new or additional resources may point to hidden barriers encountered by public health officers. This may be due to local authority budget cuts and the relative infancy of evidence-based local government interventions to tackle childhood obesity (Danielli et al., 2021).

Our research showed that operational commitment consisted of public health officer time taken to influence individuals and organizations and engage with decision-makers. Previous research has highlighted the importance of relationship-building and aligning with the priorities of others to increase commitment (Lake et al., 2017). This study highlighted specific activities including influencing the work of other departments through service plans and contracts, developing guidance documents, and providing training. Facilitating embedded commitment was linked to influencing practice rather than political commitment. However, it could be argued that working with officers from different departments could indirectly facilitate political commitment from councillors leading these portfolios. This illustrates how public health officers could span boundaries to facilitate the information flow between groups, in this case, partners and council decision-makers (Long et al., 2013).

System-wide commitment is considered a culmination of rhetorical, institutional, operational, and embedded commitment to achieve a sustained policy response (Baker et al., 2019). Researchers argue that system-wide commitment is difficult unless policy champions receive ongoing prompts and incentives from other senior officials (Pelletier et al., 2011). In our analysis, examples of sustained policy responses were not apparent, but there was evidence that progress on childhood obesity was being reported to councillors and decision-makers through Health and Wellbeing Boards and other forums. Our findings indicate that officers can proactively use local governance and democratic infrastructure to build a feedback loop of review and political oversight of health promotion policies. This ensures that the issue is appropriately framed and frequently brought to the attention of councillors and decision-makers. Although this proactive use of the governance and democratic infrastructure is necessary, alone it is unlikely to lead to system-wide commitment. Furthermore, policy scholars have observed that political attention often shifts from topic to topic, therefore this role of proactively seeking oversight is likely to be required on an ongoing basis to sustain political commitment and will require the embedding of accountability systems beyond the public health portfolio if system-wide commitment is to be achieved (Cairney, 2018).

The interviews were conducted with a modest sample of local authority officers which is a limitation of this study; however, participants provided a spread of views and a rich dataset was acquired. Gaining perspectives from multiple actors within fewer local authorities may have given greater insights. However, public health officers are key actors in the system and their perspectives are crucial for understanding political commitment and how it could be facilitated through public health practice. Qualitative data analysis was considered appropriate as the research aimed to interpret the data through the lens of political commitment and to produce actionable results (Braun & Clarke, 2021b). Prompts were used to enhance data collection, and during the analysis, regular discussions with collaborators enabled reflexive practice to strengthen this study. The role of the lead researcher, a public health practitioner, may have influenced the conduct of the study and data analysis. However, this could also be a strength in terms of understanding the roles and functions in local government. Furthermore, involving the wider supervisory team in the analysis process and undertaking the participant validation exercise minimized the possibility of any researcher bias.

Although using a framework with *a priori* themes was useful to explore the different aspects of political commitment, it may have imposed limitations on the interpretation of the data. By undertaking both deductive and inductive coding, the potential for the identification of other elements relevant to understanding political commitment to tackling childhood obesity was maximized.

The causal loop diagram illustrates how the different forms of political commitment are interrelated and it may help to identify opportunities to build political commitment. It should be considered as a starting point as it does not attempt to represent all elements that could influence political commitment in LAs.

Finally, the previous research on which this study was based largely concerned nutrition policy in low or middle-income countries. However, our study revealed valuable insights into the processes of developing local government political commitment in higher income settings to address childhood obesity. The findings from the study will help develop such commitment for health promotion policies to tackle childhood obesity.

## Implications for Policy

The study sheds light on implications for policymakers by providing evidence on the benefits of supporting public health practitioners and officers to act as policy entrepreneurs. Policymakers should proactively engage with policy entrepreneurs and support them to shape the narrative ensuring it is politically acceptable, in order to facilitate the implementation of evidence-based interventions.

## Implications for Practice

The study shows that public health practitioners and stakeholders delivering services directly to communities, should provide feedback about local issues and challenges to their managers. These insights are vital for those working with policymakers in shaping the local narrative, informing policy options and developing future services.

## Implications for Research

Public Health researchers should focus on understanding councillors’ views on local government accountability for tackling childhood obesity. Further research is needed to better understand the role that councillors could play in facilitating political commitment among themselves. Such research should use mixed methods with surveys and interviews to ensure that the required breadth and depth of data are obtained, to understand the barriers and facilitators for policy change to tackle childhood obesity in local government. Researchers should share their findings with local government policymakers and elected officials to enable them to take action to prevent childhood obesity.

## Supplemental Material

sj-docx-1-hpp-10.1177_15248399241294231 – Supplemental material for The Role of the Public Health Workforce in Securing Political Commitment for Tackling Childhood Obesity in Local Government Supplemental material, sj-docx-1-hpp-10.1177_15248399241294231 for The Role of the Public Health Workforce in Securing Political Commitment for Tackling Childhood Obesity in Local Government by Ravita Taheem, Kathyrn Woods-Townsend, Wendy Lawrence, Janis Baird, Keith M. Godfrey, Debbie Chase and Mark A. Hanson in Health Promotion Practice
