# Supplementary material for: The Role of the Public Health Workforce in Securing Political Commitment for Tackling Childhood Obesity in Local Government
Source: Health Promot Pract. 2024 Nov 18;26(5):891–901. doi: 10.1177/15248399241294231 (PMC12332212; doi:10.1177/15248399241294231)
Supplement: sj-docx-1-hpp-10.1177_15248399241294231 – Supplemental material for The Role of the Public Health Workforce in Securing Political Commitment for Tackling Childhood Obesity in Local Government [file sj-docx-1-hpp-10.1177_15248399241294231.docx]

Supplemental Material

*Table 1 Local authorities invited to participate in this study*

| **Statistical Neighbours and ONS comparators (unitary authorities) invited** | **Unitary authorities invited (South East, Public Health England, Healthy Weight Network)** | **Two Tier authorities invited (South East Public Health England Healthy Weight Network)** |
| --- | --- | --- |
| Bath North East Somerset (BNES)  Birmingham  Bournemouth  Bristol  Coventry  Derby  Kingston Upon Hull (Hull)  Leeds  Liverpool  Newcastle upon Tyne  Nottingham  Peterborough  Plymouth  Portsmouth  Salford  Sheffield  Southend on Sea  Stoke-on-Trent  York | Bracknell Forest  Brighton and Hove  Isle of Wight  Medway  Reading  Royal Borough of Windsor & Maidenhead,  Slough  West Berkshire  Wokingham | Buckinghamshire  East Sussex  Hampshire  Kent  Oxfordshire  Surrey  West Sussex |
